# Removal of Tumors without Leaving Marks

**Published:** 1881-01

**Authors:** 


### REMOVAL OF TUMORS WITHOUT LEAVING MARKS.

A writer in the *Med. Times & Gazette* removes encysted tumors in exposed localities where they are not closely adherent, by evacuating them through a small puncture, and then seizing the edges and drawing out the cyst forcibly. The design is to avoid a citatrix.

